# Supplementary material for: Environmental Factors, More Than Spatial Distance, Explain Community Structure of Soil Ammonia-Oxidizers in Wetlands on the Qinghai–Tibetan Plateau
Source: Microorganisms. 2020 Jun 21;8(6):933. doi: 10.3390/microorganisms8060933 (PMC7355592; doi:10.3390/microorganisms8060933)
Supplement: Supplementary file 1 [file microorganisms-08-00933-s001.zip › Online Resources/microorganisms-800158-1-supplementary.doc]

**Electronic Supplementary Material for:**

**Environmental Factors, More Than Spatial Distance, Explain Community Structure of Soil Ammonia-Oxidizers in Wetland on the Qinghai-Tibetan Plateau**

**Wen Zhou^1,5^, Xiaoliang Jiang^1,2^, Jian Ouyang^1,3,4^, Bei Lu^1^, Wenzhi Liu^1,5,6*^, Guihua Liu^1,5,6*^,**

*^1^ CAS Key Laboratory of Aquatic Botany and Watershed Ecology, Wuhan Botanical Garden, Chinese Academy of Sciences, Wuhan 430074, China*

*^2^ University of Chinese Academy of Sciences, Beijing 100049, China*

*^3^ Research Center for Ecology and Environment of Qinghai-Tibetan Plateau, Tibet University, Lhasa 850000, China ^3^*

*^4^ College of Science, Tibet University, Lhasa 850000, China*

*^5^Hubei Key Laboratory of Wetland Evolution & Ecological Restoration, Wuhan Botanical Garden, Chinese Academy of Sciences, Wuhan 430074, China*

*^6^Center for Plant Ecology, Core Botanical Gardens, Chinese Academy of Sciences, Wuhan 430074, China*

*The first two authors contributed equally to this work.*

*** Corresponding author：**

Email: liuwz@wbgcas.cn (W. Liu)

liugh@wbgcas.cn (G. Liu)

**Online Resource 2 Primers and conditions for qualitative and quantitative PCR**

|  | **Target gene** | **Primer set** | **Primer sequence (5'–3')** | **Size** | **Thermal profile** | **Reference** |
| --- | --- | --- | --- | --- | --- | --- |
| PCR | Archaeal amoA | Arch-amoAF | STAATGGTCTGGCTTAGACG | 635 bp | 95°C for 3 min; 40 cycles of 95°C for 15 s, 53°C for 20 s, and 72°C for 30 s; followed by a plate read at 87°C. | Francis et al., 2005. |
|  |  | Arch-amoAR | GCGGCCATCCATCTGTATGT |  |  |  |
|  | Bacterial amoA | amoA-1F | GGGGTTTCTACTGGTGGT | 491 bp |  | Rotthauweet al., 1997. |
|  |  | amoA-2R | CCCCTCKGSAAAGCCTTCTTC |  |  |  |
| Real-time PCR | Archaeal amoA | Arch-amoAF | STAATGGTCTGGCTTAGACG | 635 bp | 95°C for 3 min; 40 cycles of 95°C for 15 s, 55°C for 20 s, and 72°C for 30 s; followed by a plate read at 89°C. | Francis et al., 2005. |
|  |  | Arch-amoAR | GCGGCCATCCATCTGTATGT |  |  |  |
|  | Bacterial amoA | amoA-1F | GGGGTTTCTACTGGTGGT | 491 bp |  | Rotthauweet al., 1997. |
|  |  | amoA-2R | CCCCTCKGSAAAGCCTTCTTC |  |  |  |

References

Francis, C.A.; Roberts, K. J.; Beman, J. M.; Santoro, A. E.; Oakley, B. B. Ubiquity and diversity of ammonia-oxidizing archaea in water columns and sediments of the ocean. *Proc Natl Acad Sci USA* **2005**, 102, 14683–14688.

Rotthauwe, J. H.; Witzel, K. P.; Liesack, W. The ammonia monooxygenase structural gene amoA as a functional marker: molecular fine-scale analysis of natural ammonia-oxidizing populations. *Appl Environ Microbiol* **1997**, 63, 4704–4712.

**Online Resource 3 Spearman’s rank correlations between each environmental variable.** MAT: mean annual temperature; MAP: mean annual precipitation; Con: conductivity in water; TOC: total organic carbon in water; TN: total nitrogen in water; TP: total phosphorous in water; Stem: soil temperature; SpH: soil pH; Scon: conductivity in soil; Mois: soil moisture; STC: total carbon in soil; SNH_4_^+^: soil NH_4_^+^-N; SNO_3_^-^: soil NO_3_^-^-N; STP: total phosphorous in soil; richness: plant speices richness; coverage: plant coverage. The Spearman’s *r* and the statistical significance were shown (*** *p*< 0.001, ** *p* < 0.01, * *p* < 0.05)

**Online Resource 4** Rarefaction curves of archaeal and bacterial *amo*A OTUs (≥97% similarity) data. The lacustrine wetland data in blue, the palustrine wetland data in coral and the riverine wetland data in cyan.

**Online Resource 5** Venn diagram showing the numbers of unique and shared OTUs between three types of wetland in AOA and AOB communities. L: lacustrine wetlands, P: palustrine wetlands, R: riverine wetlands, All: all taxa, Abundant: abundant taxa.

**Online Resource 6** Maximum likelihood phylogenetic tree of archaeal (A) and bacterial (B) *amo*A gene sequences from the sediment samples. Bootstrap values above 50% of 1000 replications are indicated at branch points. The sequences obtained from this study are indicated by red circles followed by the clone names are labeled with OTU number. Accession numbers in the parentheses are followed each clone names. Numbers in the parentheses after each accession number of this study give the times that the sequence was detected in samples.

**Online Resource 7 Spearman’s correlation coefficients between the environmental factors and the diversity of AOM in Tibetan wetlands.**

|  | AOA | | | AOB | | |
| --- | --- | --- | --- | --- | --- | --- |
|  | OTU richness | Evenness | Shannon index | OTU richness | Evenness | Shannon index |
| MAT | -0.16 | 0.08 | -0.11 | -0.10 | 0.22 | -0.05 |
| MAP | **0.47**** | -0.17 | **0.48**** | 0.2 | -0.06 | 0.14 |
| Water temperature | 0.03 | 0.07 | 0.13 | 0.04 | **0.40*** | 0.19 |
| Water pH | -0.16 | -0.15 | -0.23 | 0.01 | 0.26 | 0.11 |
| Water conductivity | **-0.44*** | 0.03 | **-0.47**** | -0.44* | 0.02 | **-0.41*** |
| Water TOC | -0.06 | -0.30 | -0.17 | -0.26 | -0.09 | -0.25 |
| Water TC | -0.09 | -0.12 | -0.08 | -0.31 | -0.06 | -0.30 |
| Water TN | **-0.39*** | -0.05 | **-0.45*** | -0.29 | -0.04 | -0.29 |
| Water TP | 0.01 | -0.04 | -0.07 | -0.11 | -0.03 | -0.11 |
| Soil temperature | 0 | 0.01 | 0.02 | 0.08 | 0.28 | 0.22 |
| Soil pH | -0.11 | 0 | -0.11 | 0.04 | -0.21 | 0.03 |
| Soil conductivity | **-0.57**** | 0.33 | **-0.54**** | **-0.49**** | -0.15 | **-0.54**** |
| Soil moisture | -0.19 | 0.16 | -0.19 | 0.02 | -0.03 | -0.05 |
| Soil TC | -0.03 | -0.12 | -0.13 | -0.09 | -0.27 | -0.23 |
| Soil NH_4_^+^ | -0.21 | 0.05 | -0.19 | -0.04 | 0.33 | -0.09 |
| Soil NO_3_^-^ | 0.22 | 0 | 0.32 | -0.10 | -0.15 | -0.08 |
| Soil TP | -0.03 | 0.13 | 0 | 0.11 | 0.18 | 0.10 |
| Plant species richness | 0.21 | -0.14 | 0.24 | 0.13 | -0.19 | 0.06 |
| Plant coverage | 0.34 | -0.10 | 0.33 | 0.08 | -0.17 | 0.08 |

** *p* < 0.01; * *p* < 0.05.

The abbreviations are the same as in Online Resource 1.

**Online Resource 8 Relationship between the Euclidean distance of environmental factors and geographic distance.**
